# Glipizide sensitizes lung cancer cells to TRAIL-induced apoptosis via Akt/mTOR/autophagy pathways

**DOI:** 10.18632/oncotarget.21754

**Published:** 2017-10-09

**Authors:** Uddin MD. Nazim, Ji-Hong Moon, You-Jin Lee, Jae-Won Seol, Yong Ju Kim, Sang-Youel Park

**Affiliations:** ^1^ Biosafety Research Institute, College of Veterinary Medicine, Chonbuk National University, Iksan, Jeonbuk 54596, South Korea; ^2^ Department of Herbal Medicine Resources, College of Environmental and Bioresources, Chonbuk National University, Iksan, Jeonbuk 54596, South Korea

**Keywords:** glipizide, autophagy, TRAIL, apoptosis, lung cancer cells

## Abstract

The combination of tumor necrosis factor–related apoptosis-inducing ligand (TRAIL) with subsidiary agents is a promising anticancer strategy to conquer TRAIL resistance in malignant cells. Glipizide is a second-generation oral hypoglycemic medicine for the cure of type II diabetes because of its capability to selectively stimulate insulin secretion from β-cells. In this study, we revealed that glipizide could trigger TRAIL-mediated apoptotic cell death in human lung adenocarcinoma cells. Pretreatment with glipizide downregulation of p-Akt and p-mTOR in different concentrations. In addition, LC3-II and p-Akt was suppressed in the presence of LY294002, a well-known inhibitor of P13K. Treatment with glipizide commenced in a slight increase in conversion rate of LC3-I to LC3-II and significantly decreased p62 expression levels in a dose-dependent manner. This indicates that glipizide encouraged autophagy flux activation in human lung cancer cells. Inhibition of autophagy flux applying a specific inhibitor and genetically modified ATG5 siRNA enclosed glipizide-mediated enhancing effect of TRAIL. These data demonstrate that inhibition of Akt/mTOR by glipizide sensitizes TRAIL-induced tumor cell death through activating autophagy flux and also suggest that glipizide may be a combination therapeutic target with TRAIL protein in TRAIL-resistant cancer cells.

## INTRODUCTION

Lung cancer is the principle cause of cancer-concerned death in the world with over one million men and women diagnosed each year. Multiple options for the cure of lung cancer have been described, including radiation therapy, chemotherapy, and surgery [1, 2]. However, combination chemotherapy can be dynamic for patients with advanced cancers that are not adaptable to surgical treatment or radiation therapy.

Tumor necrosis factor (TNF)-related apoptosis-inducing ligand is a type II transmembrane cytokine. It is a member of the TNF superfamily, and mediates cellular apoptosis in a wide extent of tumor cells. However, it has little or no outcome on normal cells [3, 4]. TRAIL can bind up to five members of the death receptor family: The death receptors (DR4, DR5), the decoy receptors (DcR1, DcR2) and osteoprotegerin (OPG) [5, 6]. Of these receptors, only death receptors have cytoplasmic death domains involving in the extrinsic apoptotic pathway upon TRAIL binding [7]. TRAIL initiates apoptosis upon binding of death receptors DR4 and DR5 leads to the recruitment of Fas-associated death domain protein and consummately procaspase-8, to the construction of death-inducing signaling complex (DISC), leading to consequent effector caspases (caspase-8, -9, -10, and -3) [8, 9].

Glipizide is a second-generation oral hypoglycemic medicine developed in the 1950s for the cure of type II diabetes because of its capability to particularly stimulate insulin secretion from β-cells [10–12]. Recent studies have discovered that diabetic patients have supreme risks of developing different types of tumor [13–18]. Interestingly, epidemiological studies revealed that long-term application of some anti-diabetic drugs like as glipizide may alleviate the risk of developing cancer [19]. However, the manner in which these types of anti-diabetic drugs reduce cancer risk remains unclear.

Autophagy is a cellular self-digestion mechanism that involves degradation of unnecessary or defective cytoplasmic elements, through the actions of endogenous lysosomes, in response to converse conditions, in order to sustain cellular energy supply and homeostasis [20, 21]. Autophagy flux is the entire mechanism of autophagy, starting with the construction of autophagosomes throughout the cargo, amalgamation of the autophagosome with lysosomes, and dilapidation and recycling of the cargo [22]. Several studies have discovered that autophagy can be triggered by a diversity of stressors, such as mitochondrial loss, oxidative stress, nutrient impairment, and exogenous chemicals [23]. The most conventional appearance of autophagy, known as ‘macro-autophagy’, has been mentioned as type II programmed cell death [24]. Initiation of autophagy is negotiated by aggregation of the ULK1/2-ATG13-FIP200 compound, which results in progression of the isolation membrane, also called as phagophore which extends, and after closure, forms a vesicular composition known as the autophagosome. The role of ULK1/2-ATG13-FIP200 complex is elongation and maturation of autophagosomes [25]. The generation of this compound is coordinated by mammalian target of rapamycin, which is subsequent of the PI3K/Akt pathway. Progression of the autophagosomes contingent on class III PI3K complex, which correspond of the Vps-34, beclin1, and p150 and recruit supplementary autophagy-related proteins to allow for elongation and completion of the autophagosomes. Once the autophagosome is formed, its maturation process is complete upon amalgamation with lysosomes to form an autophagolysosome, which undergoes a cellular degradation process [23, 26]. These serine/threonine proteins are significant key regulators of many fundamental cellular systems such as cell survival, proliferation, growth, and differentiation [27]. The activation of PI3K/Akt stimulates mTOR, which encourages cells to restrain autophagy activation followed by cell death [24]. Several studies have demonstrated that autophagy promotes cancer cell death in response to multitudinous anticancer agents on apoptosis deficient cells [28–31].

The therapeutic effect of anti-diabetic drugs such as metformin as a monotherapy or in combination with TRAIL is well established [32, 33]. Therefore, the objective of this project was to determine the molecular mechanisms underlying the anticancer effect of glipizide and its synergistic outcome of glipizide combined with TRAIL in lung adenocarcinoma cells.

## RESULTS

### Glipizide sensitizes TRAIL-mediated apoptosis in lung adenocarcinoma cells

**Figure 1:**
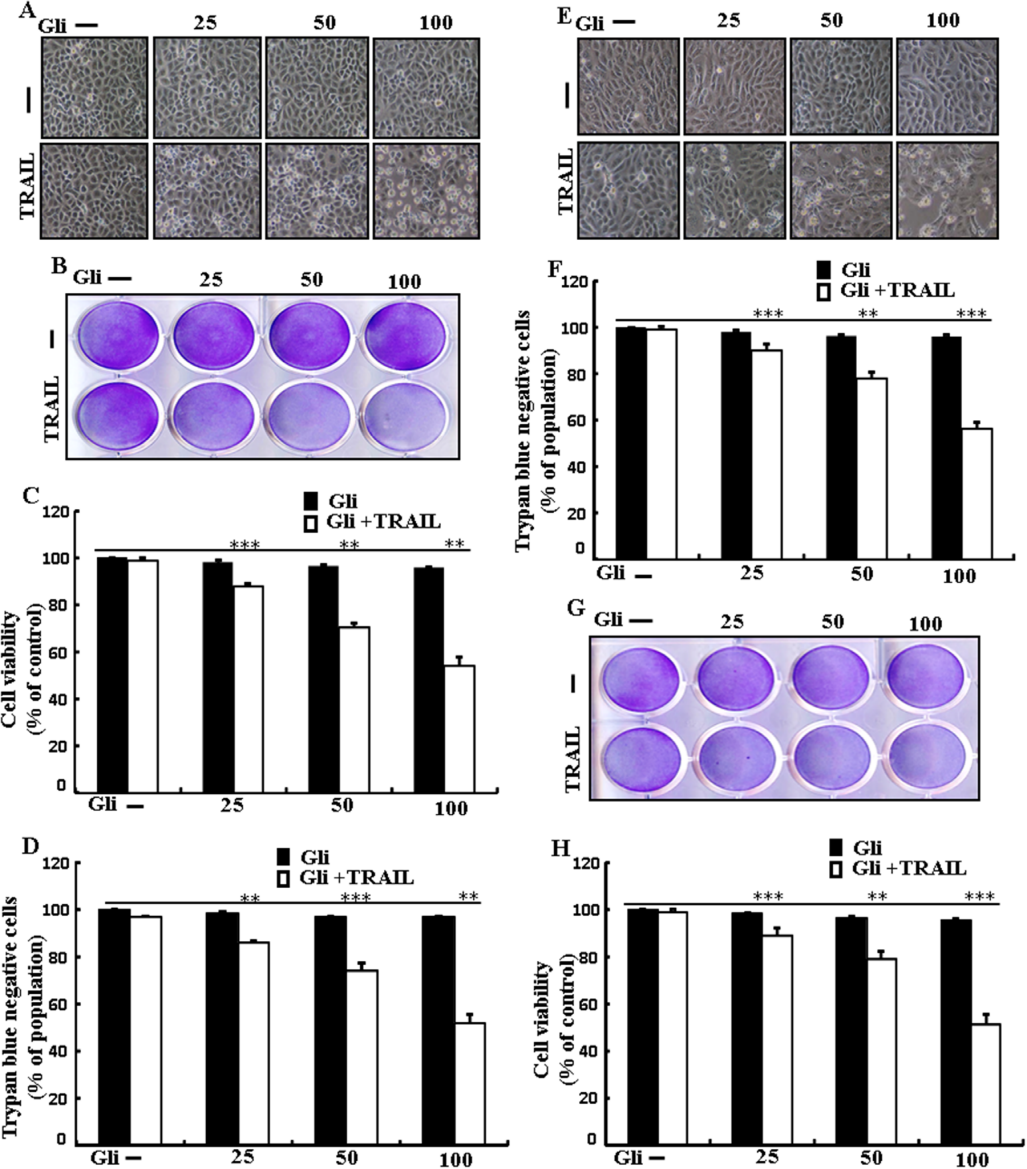
Glipizide sensitizes TRAIL-mediated apoptosis in lung adenocarcinomacells A549, HCC-15 and Calu-3 cells were pre-incubated with glipizide at different doses (0, 25, 50, and 100 μM) for 12 h and exposed to TRAIL protein 200 ng/ml for 2 h. **(A** and **E)** Cell morphology photographed using light microscope in A549 and Calu-3 Cells (×100); **(B** and **G)** Cell viability was measured with crystal violet assay in A549 andHCC-15 Cells; **(C** and **H)** Bar graph indicating the average density of crystal violet in A549 andHCC-15 Cells; **(D** and **F)** Cell viability was measured with trypan blue dye exclusion assays in A549 and Calu-3 Cells. ^**^ *p*<0.01, ^***^ p < 0.001: represent significant differences between control and each treatment group; Gli: Glipizide; TRAIL: Tumor necrosis factor (TNF)-related apoptosis-inducing ligand.

To investigate the outcome ofglipizide on TRAIL-induced apoptosis, cells were pre-incubated with varying concentrations of glipizide for 12 h and exposed to TRAIL for 2 h. Cells were photographed under a light microscope to visualize the morphological changes. Treatment of glipizide or TRAIL alone did not or only slightly influenced cell death (Figure 1) and did not morphological change was recognized compared with that in control, suggesting that A549 cells were highly resistant to TRAIL-mediated apoptosis. However, co-treatment with TRAIL and different concentrations of glipizide significantly increased the number of apoptotic cell deaths or going through apoptosis compared to glipizide or TRAIL alone (Figures 1A, 1B, 1C, and 1D). Co-treatment of TRAIL and glipizide also decreased cell viability and significantly sensitized apoptosis in Calu-3, HCC-15 cells (Figures 1E, 1F, 1G and 1H). These result suggested that glipizide sensitized TRAIL-induced apoptosis in A549, Calu-3 and HCC-15 cells.

### Glipizide induces autophagy and sensitized apoptosis mediated by TRAIL

**Figure 2:**
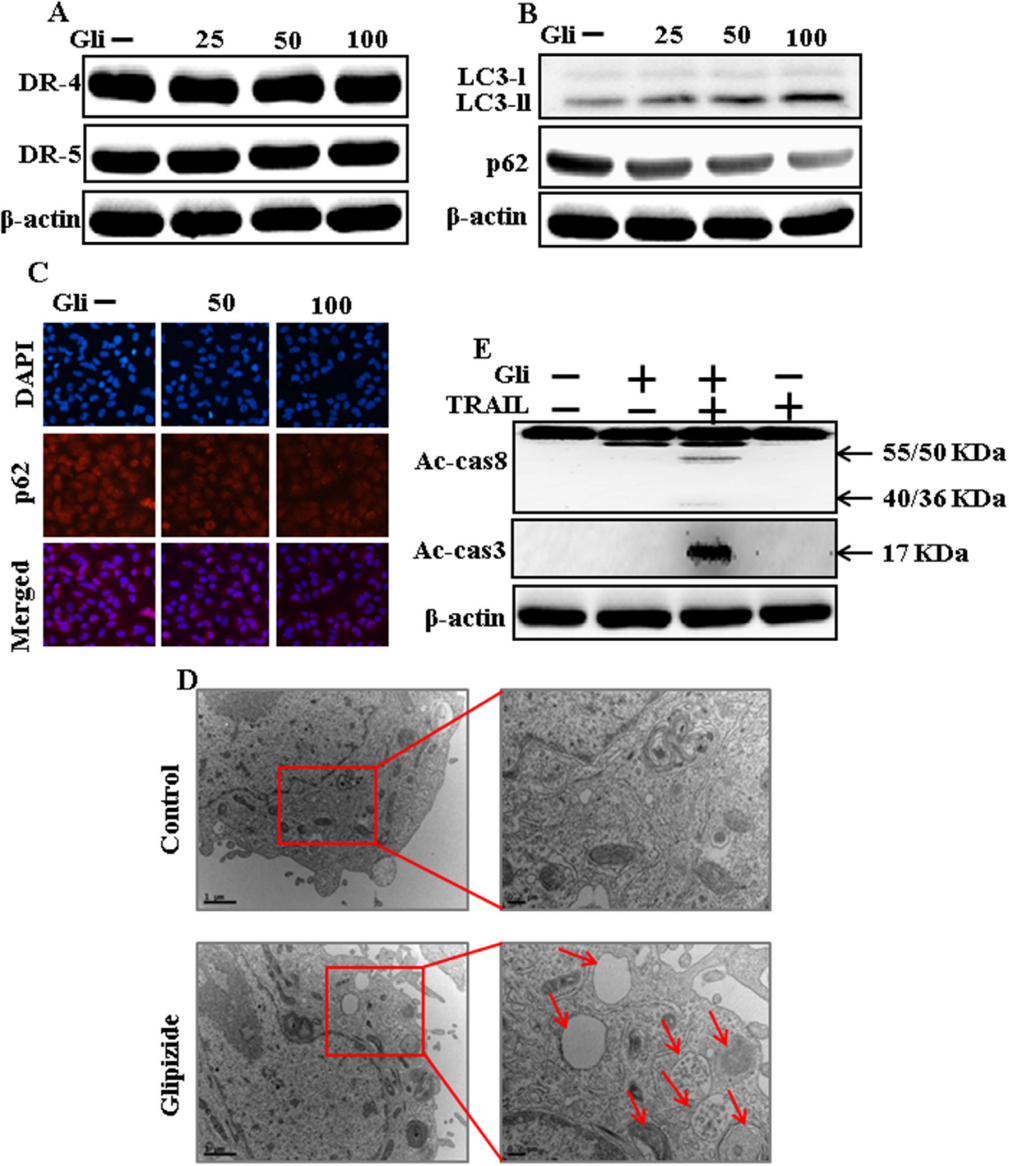
Glipizide induces autophagy and sensitized apoptosis mediated by TRAIL A549 cells were pre-incubated with glipizide at varying doses (0, 25, 50, and 100 μM) for 12 h. **(A** and **B)** Western blot for DR-4, DR-5, LC3-II, and p62 proteins was analyzed from A549 cells; **(C)** Cells were immunostained with p62 antibody (red) and observed in fluorescent view; **(D)** TEM shows the ultrastructure of cells treated with glipizide for 12 h. Arrows indicate autophagosomes, together with residual digested material and empty vacuoles; **(E)** Western blot for Ac-cas3 and Ac-cas8 expression levels was conducted with A549 cells. Cells were pre-incubated with glipizide for 12 h and exposed to TRAIL protein for an additional 1 h. β-actin was used as the loading control. Gli: Glipizide; TRAIL: Tumor necrosis factor (TNF)-related apoptosis-inducing ligand; Ac-cas3: Activated caspase 3; Ac-cas8: Activated caspase 8.

To investigate the effect of glipizide on autophagy flux. Whole cell lysates were included to western blot analysis. As shown in Figure 2A, the protein levels of DR4 and DR5 were unchanged by glipizide at varying concentrations. The formation of the autophagosome is negotiated by the Atg12-Atg5-Atg16 complex and LC3-I-phospholipid links LC3-II. P62 is an important autophagy substrate that is incorporated into autophagosomes by exactly interacting with LC3 and is ability degraded by autophagy. Inhibiting autophagy results in prompt accumulation of p62, whereas suppressed p62 levels are amalgamated with autophagy activation. Nevertheless, LC3-II increased, and p62 expression decreased after glipizide treatment in a dose-dependent manner (Figure 2B). Immunocytochemistry results also supported that various concentrations of glipizide decreased p62 protein levels (Figure 2C). A TEM assay suggested that numerous autophagic vacuoles and empty vacuoles were appeared in the cells treated with glipizide (Figure 2D). The combined treatment with glipizide and TRAIL enhanced intracellular apoptosis indicators Ac-cas3 and Ac-cas8 expression levels compare with the single treatment with TRAIL or glipizide (Figure 2E). These results reveal that glipizide can induce autophagy in A549 cells.

### Glipizide enhanced TRAIL-induced apoptosis is blocked by inhibition of autophagy

**Figure 3:**
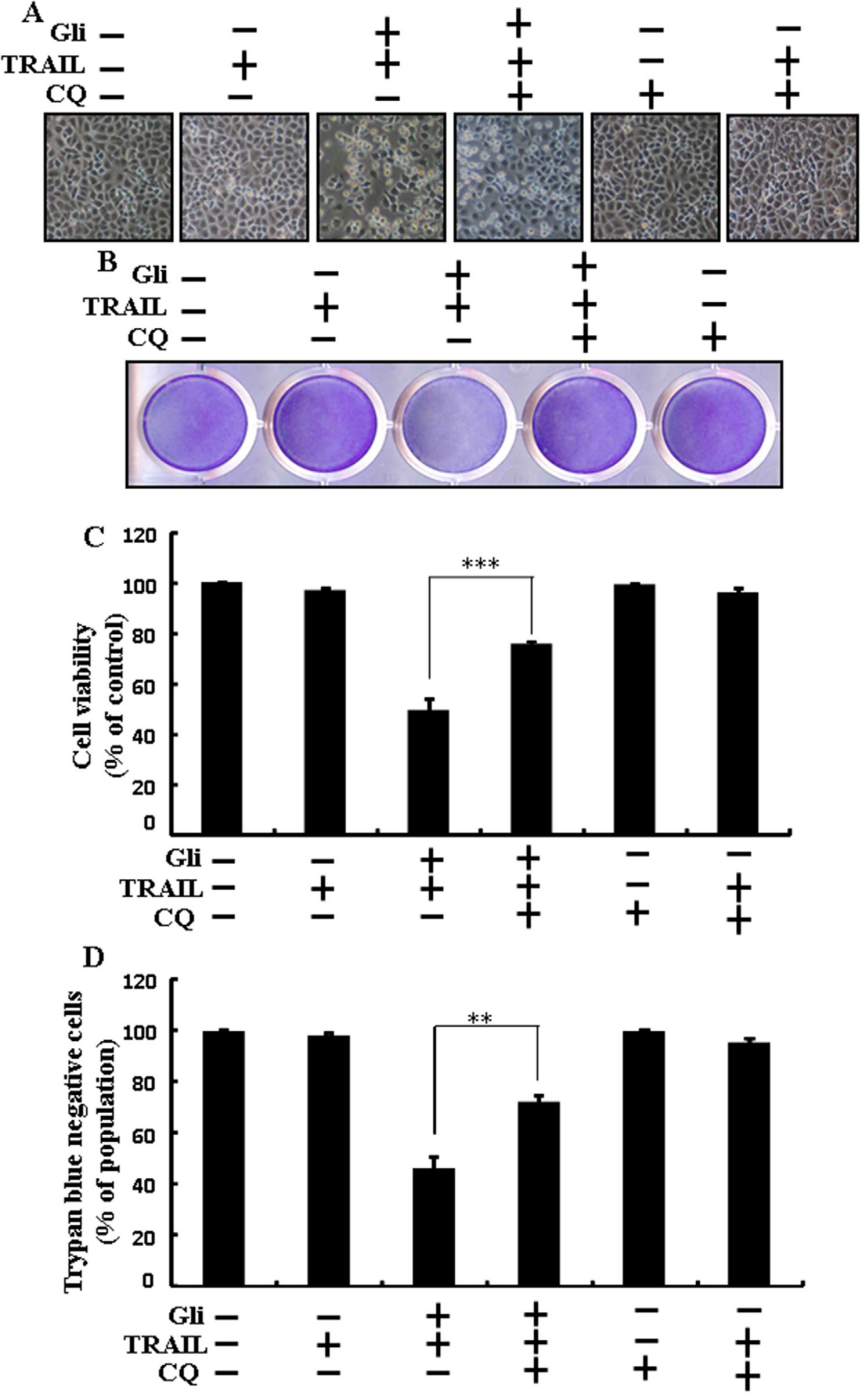
Glipizide enhanced TRAIL-induced apoptosis is blocked by inhibition of autophagy Cells were pre-incubated with the indicated glipizide doses for 12 h and exposed to TRAIL protein for an additional 2h. Additional cells were also pre-incubated with autophagy inhibitor chloroquine for 1 h followed by glipizide treatment. **(A)** Cell morphology photographed using light microscope (×100); **(B)** Cell viability was measured with crystal violet assay; **(C)** Bar graph indicating average density of crystal violet; **(D)** Cell viability was measured with trypan blue dye exclusion assays. ^**^ *p*<0.01, ^***^ p < 0.001: represent significant differences between control and each treatment group; Gli: Glipizide; TRAIL: Tumor necrosis factor (TNF)-related apoptosis-inducing ligand; CQ: Chloroquine.

Chloroquine was used to investigate the effect of glipizide on TRAIL-induced apoptosis. A549 cells were pre-incubated with the indicated glipizide concentrations for 12 h and exposed to TRAIL for 2h. Additional cells were also pre-incubated with chloroquine for 1 h, followed by glipizide. Co-treatment with TRAIL, glipizide, and chloroquine blocked cell death. However, Cell morphology results also supported that chloroquine enclosed the cell death effect compared to treatment with glipizide and TRAIL (Figure 3A). Co-treatment with TRAIL, glipizide, and chloroquine strongly increased cell viability in A549 cells with significantly decreased cell death (Figures 3B, 3C, and 3D). These data suggested that chloroquine could promote glipizide-mediated cancer cell survival induced by TRAIL.

### Autophagy inhibitor blocks TRAIL mediated apoptosis by glipizide via activating autophagy flux

**Figure 4:**
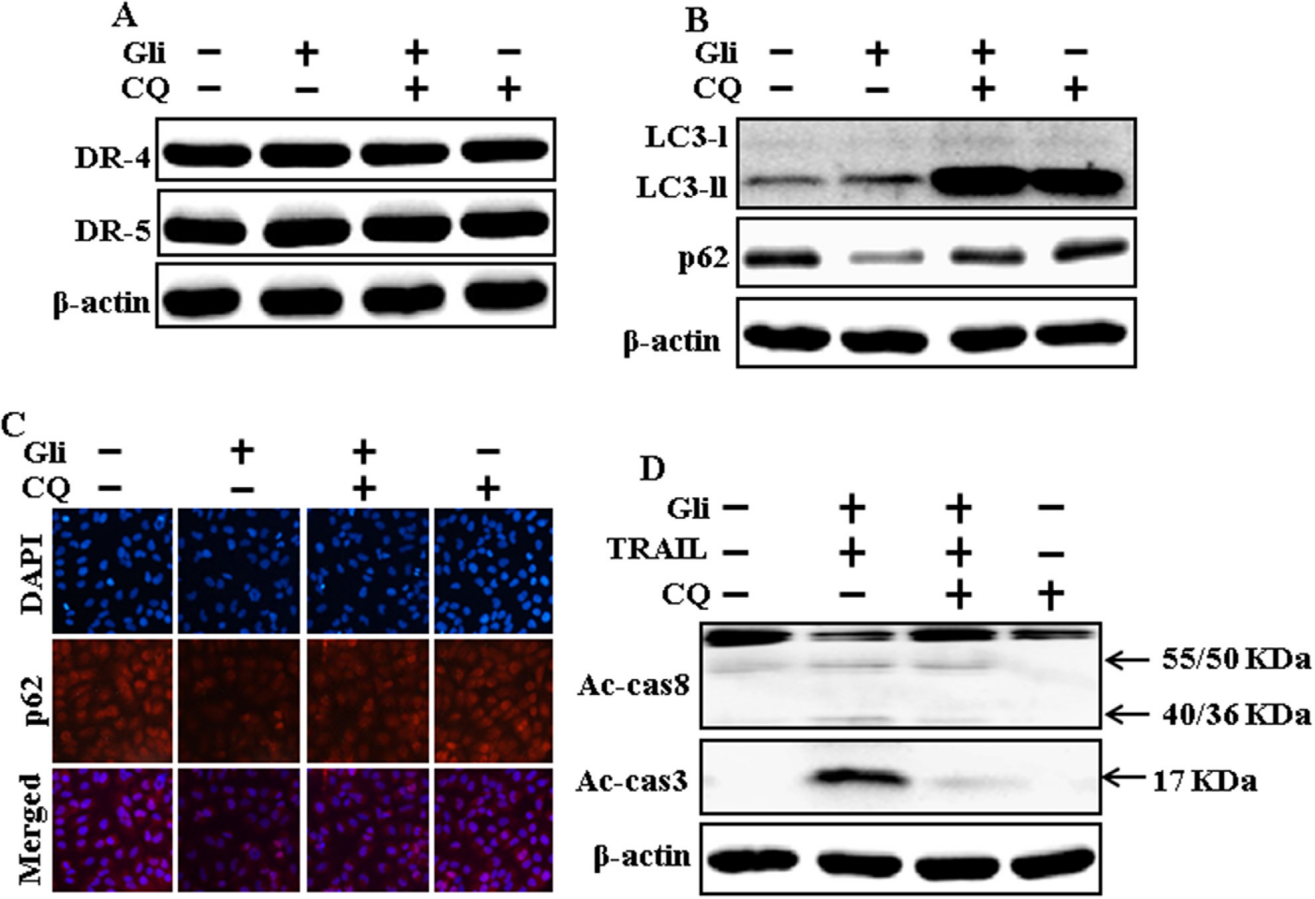
Autophagy inhibitor blocks TRAIL mediated apoptosis by glipizide via activating autophagy flux A549 cells were pre-incubated with chloroquine for 1h followed by indicated glipizide doses for 12 h. **(A** and **B)** Western blot for DR-4, DR-5, LC3-II, and p62 proteins was analyzed from A549 cells; **(C)** Cells were immunostained with p62 antibody (red) and observed in fluorescent view; **(D)** Western blot for Ac-cas3 and Ac-cas8 expression levels was conducted with A549 cells. Cells were pre-incubated with the indicated glipizide concentrations for 12 h and exposed to TRAIL protein for an additional 1h. Additional cells were pre-incubated with autophagy inhibitor chloroquine for 1 h, followed by glipizide treatment. β-actin was used as the loading control. Gli: Glipizide; Tumor necrosis factor (TNF)-related apoptosis-inducing ligand; Ac-cas3: Activated caspase 3; Ac-cas8: Activated caspase 8; CQ: Chloroquine.

We determine the effect of the glipizide on TRAIL induction of the apoptotic way by activating autophagy flux with pharmacological autophagy inhibitor chloroquine. All the cell lysates were included to western blot analysis. The expression levels of DR4 and DR5 were unchanged by chloroquine or glipizide alone or by combined treatment with chloroquine and glipizide in A549 cells (Figure 4A). Autophagy induction was further adopted by the observation of autophagic flux using chloroquine. Autophagy inhibitor Chloroquine caused impressed accumulation of membrane-bound LC3-II levels, with decreasing p62 (Figure 4B). Immunocytochemistry results also supported that glipizide treatment decreased the p62 protein level compared with chloroquine or by treatment with both glipizide and chloroquine (Figure 4C). The combined treatment of glipizide and TRAIL enhanced intracellular apoptosis indicators Ac-cas3 and Ac-cas8 expression levels. However, co-treatment of glipizide, TRAIL, and chloroquine enclosed the increase in expression level of Ac-cas3 and Ac-cas8 (Figure 4D). These results suggested that glipizide-mediated enhancement of the TRAIL-induced apoptosis could be blocked by chloroquine via activation of autophagy flux.

### Glipizide enhanced TRAIL-induced apoptosis is blocked by genetic inhibition of autophagy

**Figure 5:**
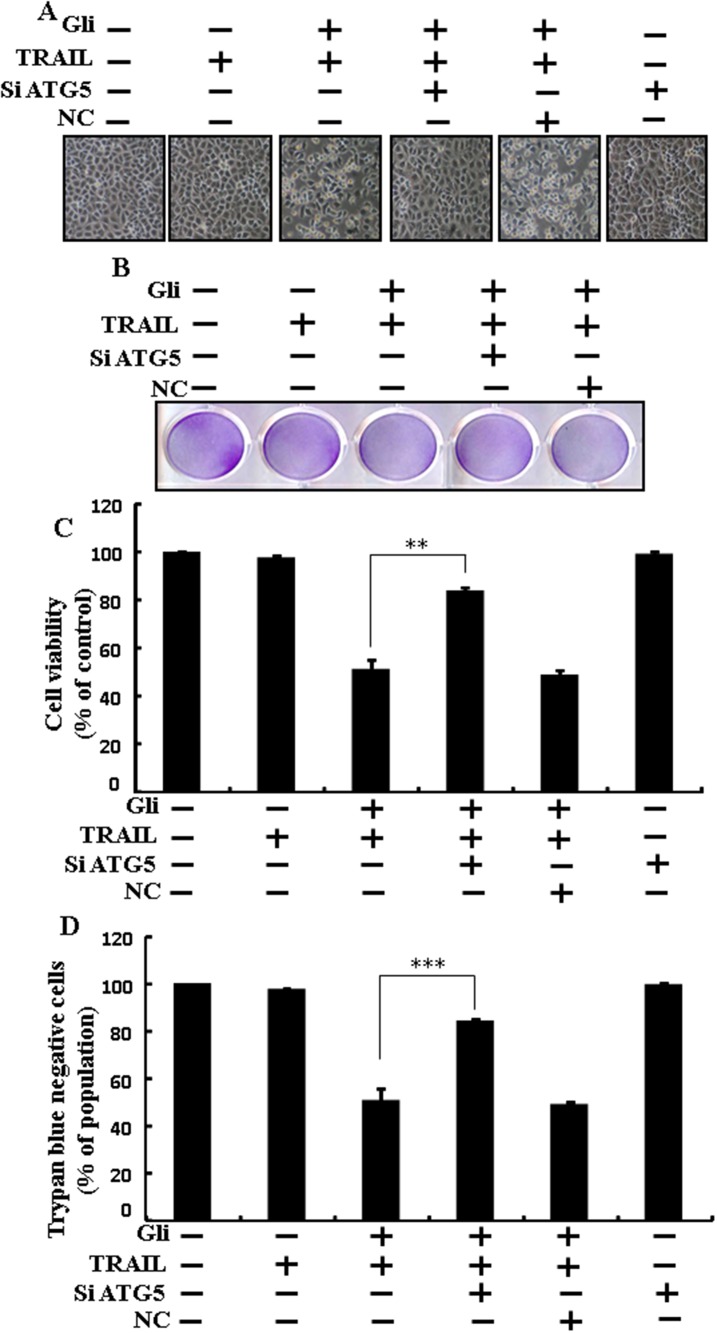
Glipizide enhanced TRAIL-induced apoptosis is blocked by genetic inhibition of autophagy A549 cells were pre-incubated with ATG5 siRNA or negative control siRNA for 24 h and then exposed to indicated glipizide doses for 12 h with or without TRAIL protein for an additional 2 h. **(A)** Cell morphology photographed using light microscope (×100); **(B)** Cell viability was measured with crystal violet assay; **(C)** Bar graph indicating average density of crystal violet; **(D)** Cell viability was measured with trypan blue dye exclusion assays. ^**^ *p*<0.01, ^***^ p < 0.001: represent significant differences between control and each treatment group. Gli: Glipizide; TRAIL: Tumor necrosis factor (TNF)-related apoptosis-inducing ligand; siATG5: ATG5 small interfering RNA; NC: Negative control.

Genetic autophagy inhibitor ATG5 siRNA used to determine the effect of glipizide on TRAIL-induced apoptosis. A549 cells were pre-incubated with ATG5 siRNA or NC for 24 h and then exposed to indicate glipizide doses for 12 h with or without TRAIL for 2 h. Co-treatment of glipizide, ATG5 siRNA, and TRAIL blocked cell death. However, Cell morphology results also supported that ATG5 siRNA blocked cell death effect compared to glipizide, TRAIL, and negative control siRNA treatment (Figure 5A). Co-treatment with glipizide, TRAIL, and ATG5 siRNA strongly increased cell viability in A549 cells with significantly decreased cell death (Figure 5B, 5C, and 5D). These results suggested that ATG5 siRNA could promote glipizide-mediated cancer cell survival induced by TRAIL.

### Genetic autophagy inhibitor blocks TRAIL-induced apoptosis by glipizide via activation of autophagic flux

**Figure 6:**
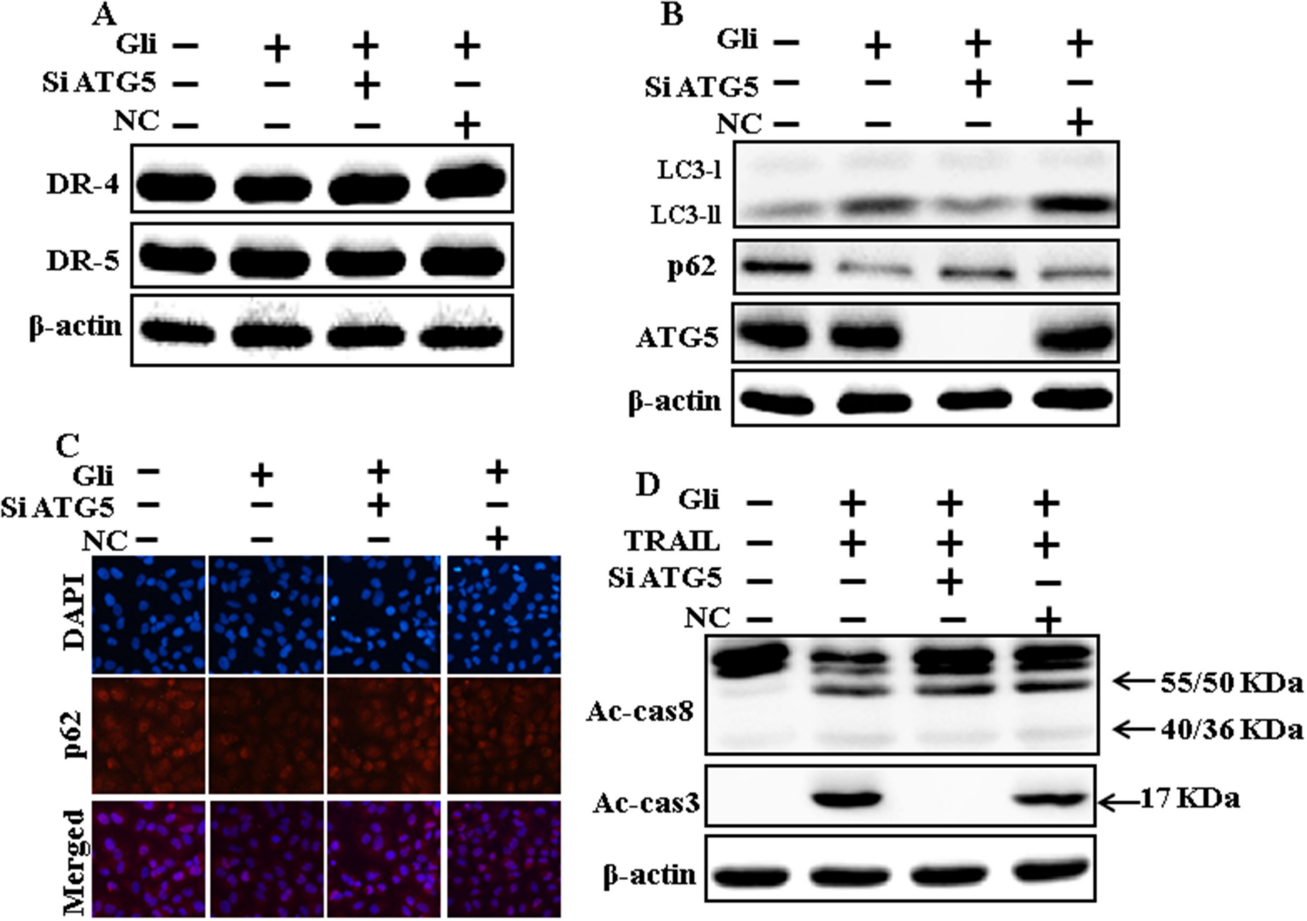
Genetic autophagy inhibitor blocks TRAIL-induced apoptosis by glipizide via activation of autophagic flux A549 cells were pre-incubated with ATG5siRNA or negative control siRNA for 24 h, and then exposed to indicated glipizide doses for 12 h. **(A** and **B)** Western blot for DR-4, DR-5, LC3-II, p62 and ATG5 proteins was analyzed from A549 cells; **(C)** Cells were immunostained with p62 antibody (red) and observed in fluorescent view; **(D)** Western blot for Ac-cas3 and Ac-cas8 expression levels was conducted. A549 cells were pre-incubated with ATG5siRNA or negative control siRNA for 24 h, and then exposed to indicated glipizide doses for 12 h with or without TRAIL protein for an additional 1 h. β-actin was used as the loading control. Gli: Glipizide; TRAIL: Tumor necrosis factor (TNF)-related apoptosis-inducing ligand; Ac-cas3: Activated caspase 3; Ac-cas8: Activated caspase 8; siATG5: ATG5 small interfering RNA; NC: Negative control.

We determine the effect of the glipizide-induced TRAIL-mediated apoptotic pathway by activating autophagy flux withgenetic autophagy inhibition by ATG5 siRNA. All the cell lysates were included to western blot analysis. The expression levels of DR4 and DR5 were unchanged by glipizide alone or by combined treatment with ATG5 siRNA or NC in A549 cells (Figure 6A). To address the induction of autophagy, cells were transfected with siRNA directed in opposition to autophagy protein 5 (Atg5) to block autophagic vesicle composition, and silencing of ATG5 was confirmed. Knockdown of ATG5 markedly decreased the glipizide-induced LC3-II protein level (Figure 6B). Immunocytochemistry results also suggested this p62 protein level in A549 cells (Figure 6C). Co-treatment of glipizide, NC siRNA, and TRAIL enhanced intracellular apoptosis indicators Ac-cas3 and Ac-cas8. Nevertheless, co-treatment with glipizide, ATG5 siRNA, and TRAIL enclosed the increase in Ac-cas8 and Ac-cas3 expression levels (Figure 6D). These results suggested that glipizide-mediated enhancement of the TRAIL-induced apoptosis could be blocked by genetic inhibition of autophagy via activation of autophagy flux.

### Effects of glipizide on the Akt/mTOR/autophagy signaling pathway

**Figure 7:**
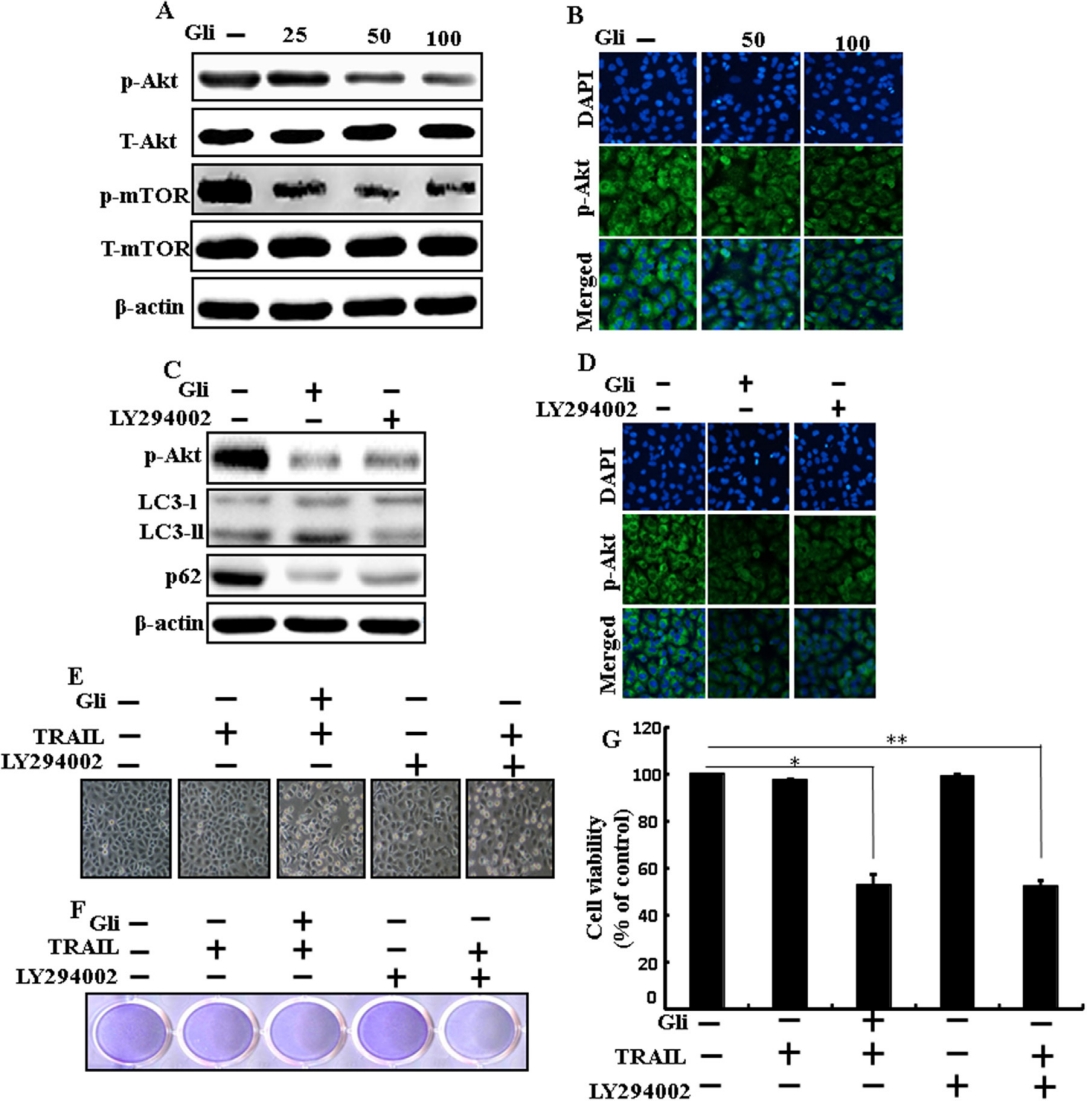
Effects of glipizide on the Akt/mTOR/autophagy signaling pathway Lung adenocarcinoma cells were pre-incubated with different doses of glipizide (0, 25, 50, and 100 μM) for 12 h and exposed to TRAIL protein for an additional 2h. Additional cells were pretreated with LY294002 for 1 h followed by treatment with glipizide. After that, **(A** and **C)** western blot for T-Akt, p-Akt, p-mTOR, T-mTOR, LC3-II, and p62 proteins was analyzed from A549 cells; **(B** and **D)** Cells were immunostained with p-Akt antibody (green) and observed in fluorescent view; **(E)** Cell morphology photographed using light microscope (×100); **(F)** Cell viability was measured with crystal violet assay; **(G)** Bar graph indicating average density of crystal violet. β-actin was used as loading control. ^*^p < 0.05, ^**^p < 0.01: represent significant differences between control and each treatment group; Gli: Glipizide; TRAIL: Tumor necrosis factor (TNF)-related apoptosis-inducing ligand.

We determine the outcome of glipizide on the Akt/mTOR pathway. Pretreatment of glipizide inducedinhibition of p-Akt and p-mTOR in a dose-dependent manner (Figure 7A). Immunocytochemistry results also supported that various concentrations of glipizide decreased p-Akt protein levels (Figure 7B). Western blot analyses revealed that LC3-II and p-Akt was suppressed in the presence of LY294002 (Figure 7C). ICC results also supported that p-Akt were inhibited in the presence of LY294002 (Figure 7D Morphological image and crystal violet staining results display that combined treatment with LY294002 and TRAIL decreased cell viability and significantly sensitized apoptosis in A549 cells, similar to treatment with TRAIL and glipizide (Figures 7E, 7F, and 7G). These results suggest that the role of glipizide function is not only based on suppression of the pathway but is also contingent on the induction of autophagy.

## DISCUSSION

The purpose of this project was to determine the effect of glipizide with or without TRAIL on lung adenocarcinoma A549 cells. Our results demonstrated that glipizidesensitizes human lung cancer cells to TRAIL-mediated apoptosis via Akt/mTOR/autophagy pathways.

TRAIL could be a safe and dynamic biological candidate that can be utilized for tumor therapy in humans. It has recently accomplished significant interest in medical knowledge, as it can selectively induce tumor cells, virus-infected cells, and transformed cells to maintain apoptosis without harming toxicity in normal cells [34–38]. Recent pharmacoepidemiological surveys report that the treatment of antidiabetic drugs can attribute cancer risk in patients with type 2 diabetes. It was also revealed that diabetic patients prescribed with glipizide are at lower hazard of developing cancer [39]. Autophagy is a lysosome-dependent degradation process activated by starvation, hypoxia, growth inducing factor distress, or endoplasmic reticulum stress [40]. Consequently, autophagy plays a critical role in the degeneration of cytoplasmic proteins and other macromolecules by disintegrating damaged or aged organelles [41, 42]. Recent studies suggest that inhibition of the PI3K/Akt signaling pathway and its downstream goal mTOR initiates autophagy [43]. Accordingly, the suppression of the class I PI3K/Akt/mTOR pathway is an imperious and attractive target for cancer therapy.

Jin *et al.* [44] demonstrated that A549 cells are resistant to TRAIL. In our present study, we also observed that single treatment of glipizide or TRAIL had negligible effects on apoptosis in A549 cells. Thus, scientists are currently tempting to identify TRAIL sensitizers that are proficient at overcoming TRAIL resistance in cancer cells. Here we show that co-treatment with TRAIL and varying concentrations of glipizide significantly increased the number of apoptotic cell deaths or going through apoptosis compared to glipizide or TRAIL alone (Figure 1). Some reports have demonstrated that some anti-diabetic drugs inhibited cancer cell proliferation as well as tumors in animal models [45]. However, our western blot and ICC results revealed LC3-II was increased and p62 was decreased after glipizide treatment in a dose-dependent manner, though co-treatment of glipizide with TRAIL enhanced intracellular apoptosis indicators Ac-cas3 and Ac-cas8 expression levels compared to treatment with TRAIL or glipizide alone (Figure 2). Our results also suggested that specific pharmacological inhibitor chloroquine promoted the survival of lung adenocarcinoma A549 cells (Figure 3 and Figure 4). In addition, genetic autophagy inhibitor blocked glipizide mediated apoptosis of A549 cells induced by TRAIL (Figure 5 and Figure 6). The PI3K/Akt/mTOR signaling pathway plays a cardinal role in the tumorigenesis of human tumors [46, 47], which makes this pathway a significant target for molecular drug therapies. Our results demonstrate that Pretreatment of glipizide inducedinhibition of p-Akt and p-mTOR in varying concentrations. Western blot analyses revealed that LC3-II and p-Akt was suppressed in the presence of LY294002 (Figure 7).

In summary, Akt/mTOR signaling pathway inhibition by glipizide sensitizes TRAIL-induced tumor cell death in A549 cells via autophagy flux. Combined treatment of glipizide with TRAIL might be an adequate therapeutic technique to carefully treat some TRAIL-resistant cancers, including lung adenocarcinoma cells.

## MATERIALS AND METHODS

### Cell culture

Cancer cells originating from human lung (A549, HCC-15 and Calu-3) tumors were obtained from the American Type Culture Collection (Global Bioresource Center, Manassas, VA, USA). Cells were maintained in RPMI-1640 (Gibco BRL, Grand Island, NY, USA) medium containing 10% fetal bovine serum and 100μg/ml penicillin-streptomycin. Cells were maintained at 37 °C and 5% CO_2_ in humidified incubator.

### Reagents

Recombinant glipizide, chloroquine, and LY294002 (PI3K inhibitor) were purchased from Sigma-Aldrich (St. Louis, MO, USA). Recombinant TRAIL (200 ng/ml) was purchased from Abfrontier (Geumcheon-gu, Seoul, South Korea).

### Cell viability analysis

A549, Calu-3 and HCC-15 cells were plated at 1.0 × 10^4^ cells onto 12-well plates and incubated at 37°C for 24 h. The cells were pre-incubated with varying concentration of glipizide (0, 25, 50, and 100 μM) for 12h and exposed to TRAIL for 2h. Additional cells were also pretreated with chloroquine (20 μM) and LY294002 (10 μM) for 1 h, followed by glipizide treatment. Cell morphology was examined by photographs taken under inverted microscopy (Nikon, Japan). Cell viability was determined applying crystal violet staining method as previously described [32].

### Trypan blue exclusion assay

The number of cell viability was examined by trypan blue dye exclusion assay (Sigma-Aldrich) using a hemocytometer. The result was mainly expressed as the percentage of viable cells compared with that of vehicle-treated controls.

### Western blot analysis

A549 cell lysates were prepared by harvesting, washing in cold PBS, resuspending in lysis buffer followed by sonication. Proteins (35 μg) were resolved by 10%–15% SDS gels and transferred to a nitrocellulose membrane, and analyzed by western blotting as described previously [48]. The following antibodies were used for immunoblotting: LC3(Novus Biologicals, Littleton, CO, USA), p62 (Millipore Corp., Milford, MA, USA), ATG5, cleaved caspase-3, mTOR (Cell Signaling Technology, Danvers, MA, USA), DR-4, DR-5, and ß-actin Sigma-Aldrich (St. Louis, MO, USA), cleaved caspase-8 (BD pharmingen, USA), p-Akt (Abcam, England).

### Immunocytochemistry

A549 cell lines cultured on glass coverslips positioned on a 24-well plate. The cells were washed with PBS and fixed with 4% paraformaldehyde for 15 min at room temperature. Following this, Cells were then washed twice with ice-cold PBS, blocked with 5% FBS inTris-buffered saline with Tween, and incubated with monoclonal antibodies against p62, p-Akt at room temperature for 24 h. Unbound antibody was removed with PBS wash ( three times) and Cells were then incubated again with secondary antibody at room temperature for 2 h in the dark. Finally, cells were mounted with DakoCytomation fluorescent mounting medium and visualized via a fluorescence microscopy.

### TEM (Transmission Electron Microscopy) analysis

TEM samples were analyzed by Transmission Electron Microscope (JEM-2010, JEOL) installed in the Center for University-Wide Research Facilities (CURF) at Chonbuk National University. After fixation of A549 cell samples in 2 % glutaraldehyde and 2 % paraformaldehyde in 0.05 sodium cacodylate buffer, specimens were post fixed in 1% osmium tetroxide, dehydrated in graded ethanol and propylene oxide. A549 cells were embedded in Epoxy resin. Ultrathin sections were cut on an LKB-III ultratome and were stained with 0.5% uranyl acetate and lead citrate. The images were taken on a Hitachi H7650 electron microscope at an accelerating voltage of 100 kV.

### RNA interference

A549 cells were transfected with ATG5-specific small interfering RNA (siRNA; oligo ID HSS114103; Invitrogen, Carlsbad, CA, USA) using Lipofectamine 2000 according to the manufacturer’s instructions. After 36-h post transfection, the knockdown efficiency at protein level was observed by immunoblotting and cell viability test. Nonspecific siRNA was used as a negative control.

### Statistical analysis

All data are expressed as means ± standard deviation (SD) and were compared using the Student’s *t*-test, analysis of variance and the ANOVA Duncan test using SAS statistical package (SAS Institute, Cary, NC, USA). Statistical significance was indicated by a *P* value less than 0.05 (^*^), 0.01 (^**^), or 0.001 (^***^).
